# Overview of Extracellular Vesicles, Their Origin, Composition, Purpose, and Methods for Exosome Isolation and Analysis

**DOI:** 10.3390/cells8070727

**Published:** 2019-07-15

**Authors:** Laura M. Doyle, Michael Zhuo Wang

**Affiliations:** Department of Pharmaceutical Chemistry, School of Pharmacy, University of Kansas, Lawrence, KS 66047, USA

**Keywords:** exosomes, isolation, analysis, exosomal marker protein, microvesicles

## Abstract

The use of extracellular vesicles, specifically exosomes, as carriers of biomarkers in extracellular spaces has been well demonstrated. Despite their promising potential, the use of exosomes in the clinical setting is restricted due to the lack of standardization in exosome isolation and analysis methods. The purpose of this review is to not only introduce the different types of extracellular vesicles but also to summarize their differences and similarities, and discuss different methods of exosome isolation and analysis currently used. A thorough understanding of the isolation and analysis methods currently being used could lead to some standardization in the field of exosomal research, allowing the use of exosomes in the clinical setting to become a reality.

## 1. Introduction

Extracellular vesicles (EVs) are lipid bound vesicles secreted by cells into the extracellular space [1,2]. The three main subtypes of EVs are microvesicles (MVs), exosomes, and apoptotic bodies, which are differentiated based upon their biogenesis, release pathways, size, content, and function [1,2,3]. The content, or cargo, of EVs consists of lipids, nucleic acids, and proteins—specifically proteins associated with the plasma membrane, cytosol, and those involved in lipid metabolism [1,4]. The primary focus of this review will be on the protein content of EVs, however, the nucleic acid and lipid composition of EVs is well described in [1,2,5] and [6,7,8], respectively. While no specific protein markers have been identified to distinguish between the different types of EVs, MVs, exosomes, and apoptotic bodies have different protein profiles due to their different routes of formation [9,10,11]. However, substantial overlap of protein profiles is often observed, due in part to the lack of standardized isolation and analysis methods of EVs [2,12]. Further, it has been demonstrated that the proteomic profiles of EVs from the same source are dependent on their isolation method [2]. The field of EVs has led to much understanding in the area of cell–cell communication and cancer metastasis, and their use in the clinical setting as carriers of biomarkers for diagnostic purposes has been demonstrated [13,14,15,16,17,18,19,20,21,22,23,24,25,26,27,28], however, standardized methods for EV isolation and analysis must be developed in order for them to become tools that can truly be used in the clinical setting.

### 1.1. Exosomes

Exosomes, also referred to as intraluminal vesicles (ILVs), are enclosed within a single outer membrane, and are secreted by all cell types and have been found in plasma, urine, semen, saliva, bronchial fluid, cerebral spinal fluid (CSF), breast milk, serum, amniotic fluid, synovial fluid, tears, lymph, bile, and gastric acid [18,22,23,24,29,30,31,32,33,34,35,36,37,38,39]. 

#### 1.1.1. Origin and Size

Exosomes are a subtype of EV formed by an endosomal route and are typically 30–150 nm in diameter [1,3,4,5]. Specifically, exosomal vesicles form by inward budding of the limiting membrane of early endosomes, which mature into multivesicular bodies (MVBs) during the process [2,4,5]. Early endosomes, which originate from inward budding of the cell’s plasma membrane, and MVBs are involved in the endocytic and trafficking functions of the cell’s material [3]. Specifically, they are involved in protein sorting, recycling, storage, transport, and release [3]. MVBs are eventually either sent to the lysosome to be degraded along with all of its components or fused with the cell’s plasma membrane to release its content, including exosomes, into the extracellular space [4,40,41,42]. The factors that determine the fate of a specific MVB are not well understood [5]. However, studies have been done to demonstrate that the fate of a particular MVB depends on the level of cholesterol in the MVB. Specifically, a cholesterol rich vesicle was secreted while a morphologically identical vesicle that lacked cholesterol was sent to the lysosome for degradation [43]. The regulation of MVB and exosome formation and release is through the endosomal sorting complexes required for transport (ESCRT) pathway [44,45]. While the exact mechanism is still not fully understood, it appears the formation of MVBs can be stimulated by growth factors and the cell adjusts its exosome production according to its needs [29,46].

#### 1.1.2. Composition

The biogenesis of exosomes can be used to understand the proteome of the vesicles. Because exosomal formation and MVB transportation are regulated by ESCRT proteins, these proteins and its accessory proteins (Alix, TSG101, HSC70, and HSP90β) are expected to be found in exosomes regardless of the type of cell from which they originate [10,47,48,49,50]. Thus, this set of proteins are often termed “exosomal marker proteins.” Some studies indicate there is another mechanism, an ESCRT independent mechanism, by which some cells release exosomes into the extracellular space [51]. In such cases, exosome release is thought to depend on sphingomyelinase enzyme instead of ESCRT, since cells depleted of the ESCRT machinery still produced CD63 positive exosomes [52,53,54,55]. The CD63, along with CD9 and CD81, are proteins in the tetraspanin family. These transmembrane proteins, and other proteins associated with the plasma membrane, are commonly found in exosomes and are often enriched in the vesicles compared to the cell lysate [56,57]. Originally, it was thought that tetraspanin proteins were specific markers of exosomes, however, these proteins have since been identified in MVs and apoptotic bodies [58,59]. Exosomes tend to be enriched in glycoproteins compared to the secreting cells, however, MVs (discussed in Section 1.2) are thought to contain proteins with higher levels of posttranslational modifications (PTMs), such as glycosylation and phosphorylation, compared to exosomes, which is a potential way to distinguish the vesicles based on content rather than size [12,57,60]. Finally, a recent study [61] reported interorganelle trafficking between mitochondria and the endolysosomal system, which challenges the untested dogma that proteins specifically associated with organelles such as mitochondria and the nucleus are not expected to be observed in the exosomal vesicles. Proteins associated with the Golgi apparatus and endoplasmic reticulum, however, are thought to be present at low levels since early endosomes can interact with these organelles. Nonetheless, such proteins are typically still considered to be non-exosomal marker proteins since they are at lower levels in the exosomes compared to the lysate.

#### 1.1.3. Biological Purpose

Exosomes were originally thought to be a source of cellular dumping, or a way for cells to get rid of unneeded or unwanted material, however, it has since been found that exosomes participate in cell–cell communication, cell maintenance, and tumor progression, as discussed in Section 1.2.3. In addition, exosomes have been found to stimulate immune responses by acting as antigen-presenting vesicles [62,63]. In the nervous system, exosomes haven been found to help promote myelin formation, neurite growth, and neuronal survival, thus playing a role in tissue repair and regeneration [64,65,66,67,68]. At the same time, exosomes in the central nervous system (CNS) have been found to contain pathogenic proteins, such as beta amyloid peptide, superoxide dismutase, and alpha synuclein that may aid in disease progression [69,70,71,72]. 

#### 1.1.4. Applications and Uses

A common interest in exosomal research is in studying their ability to act as carriers of biomarkers for diseases. For example, exosomes in both plasma and CSF have been found to contain alpha synuclein, a protein associated with Parkinson’s disease [73,74,75]. A recent review has focused on EVs as markers of glioblastoma [76]. Exosomes isolated from urine have demonstrated the ability to reflect acute kidney injury [14]. There has also been success in finding markers for pancreatic cancer and lung cancer in exosomes as well [28,77]. The use of exosomes as carriers of biomarkers is ideal because these vesicles are found in bodily fluids, such as blood and urine, which allows for minimally to non-invasive “liquid biopsy” type methods to diagnose and even monitor a patient’s response to treatment. The ability of exosomes to monitor a patient’s response is yet another potential application of these vesicles in the clinical setting [13]. If the disease marker directly correlates to disease state, and if the patient’s treatment is working, one should observe a change in the presence of the biomarker as the patient undergoes treatment. Others have suggested that exosomes can be used in vaccine development and for other immunological purposes [62,63]. Because exosomes act inherently as antigen presenting vesicles, it may be possible to capitalize on this inherent property. Further, exosomes have a long circulating half-life, are well tolerated by the human body, and capable of not only penetrating cellular membranes but also potentially targeting specific cell types, which makes them an even better candidate for such immunological applications [78]. Also, because of these inherent advantages of exosomes, they are also ideal for the development of drug delivery systems [79]. While methods are still being developed for introduction of RNA and protein to exosomes, and to target these exosomes to a specific region of the body, the ability to load both protein and genetic material into exosomes is yet another advantage making exosomes an attractive drug delivery system [78]. Finally, it has been demonstrated that the mesenchymal stem cell exosomes themselves can act as a therapeutic entity to help reduce tissue injury [80,81,82,83,84]. While there is a broad range of potential applications and uses of exosomes in the clinical setting, more standardized methods for exosome isolation and analysis are needed in order to meet the regulatory requirements of the FDA and other regulatory agencies to use exosomes as biomarkers, vaccines, drug delivery devices, and therapeutic tools [5]. 

### 1.2. Microvesicles

#### 1.2.1. Origin and Size

MVs are EVs that form by direct outward budding, or pinching, of the cell’s plasma membrane. The size of MVs typically range from 100 nm up to 1 μm in diameter [1,2,3,4,5]. The route of MV formation is not well understood, however, it is thought to require cytoskeleton components, such as actin and microtubules, along with molecular motors (kinesins and myosins), and fusion machinery (SNAREs and tethering factors) [85]. The number of MVs produced depends on the donor cell’s physiological state and microenvironment [1]. Likewise, it has been previously demonstrated that the number of MVs consumed depends on the physiological state and microenvironment of recipient cells [1]. Further, the uptake of MVs is likely an energy dependent process, as uptake is suppressed at lower temperatures [60,86,87]. 

#### 1.2.2. Composition

While the proteomic profiles of MVs are heavily dependent on the isolation method, there is a category of proteins termed “marker proteins”, which are proteins found in MVs, regardless of cell origin, as a result of their biogenesis process [88]. Because MVs form by an outward budding of the cell’s plasma membrane, it is easily understood that MVs contain mainly cytosolic and plasma membrane associated proteins, especially proteins known to cluster at the plasma membrane surface, such as tetraspanins [89,90]. It has been reported that such proteins can have 100-fold higher concentration in MVs compared to the cell lysate [89,90]. Other proteins commonly identified in MVs include cytoskeletal proteins, heat shock proteins, integrins, and proteins containing post translational modifications, such as glycosylation and phosphorylation [91,92,93]. Interestingly, the glycan binding proteins on the surface of MVs maybe a key factor in understanding how MVs are targeted to, and interact with, other cells. The focus of this review will remain on the proteome of MVs, however, the glycome of MVs is thoroughly discussed in [2]. The presence of cytosolic and plasma membrane proteins can be understood based on the biogenesis of MVs, similarly, it can be understood that proteins specifically associated with different organelles such as the mitochondria, Golgi apparatus, nucleus, and endoplasmic reticulum should be depleted in MVs, especially compared to the cell’s lysate, as these organelles are not involved in the biogenesis of MVs [57,87]. However, specific markers are still lacking to distinguish MVs from exosomes [11].

#### 1.2.3. Biological Purpose

Originally, it was thought that, like exosomes, MVs were a cellular dumping or maintenance mechanism, by which the cell would get rid of unwanted material [2]. However, it has since been understood that MVs (and exosomes) are involved in cell–cell communication between local and distant cells. The ability of these EVs to alter the recipient cell has been well demonstrated [94,95]. These new discoveries in biological purpose of EVs have spurred a global interest in fully understanding EVs and the diagnostic and therapeutic potential. Other forms of cell–cell communication, such as hormones, growth factors, cytokines, and direct interaction are better understood and play an important role as to how multi-cellular organisms are able to function as a single system [2]. The uniqueness of EVs is that they have the ability to package active cargo (proteins, nucleic acids, and lipids) and deliver it to another cell, neighboring or distant, and alter the recipient cell’s functions with its delivery [1]. While such forms of communication occur between physiologically healthy cells, one could understand that diseased cells, such as cancer cells, package their active machinery in EVs, transport it to otherwise healthy cells, thus playing a role in cancer metastasis [96,97]. Perhaps a better understanding of MV and exosomal formation and regulation could lead to new options for cancer therapies, since they appear to play a critical role in cancer development and progression. 

#### 1.2.4. Applications and Uses

The applications of and uses of MVs in the clinical setting are similar to those of exosomes (1.1.4).

### 1.3. Apoptotic Bodies

#### 1.3.1. Origin and Size

Apoptotic bodies are released by dying cells into the extracellular space. They are reported to range in size from 50 nm up to 5000 nm in diameter, with the size of most apoptotic bodies tending to be on the larger side [3]. These bodies form by a separation of the cell’s plasma membrane from the cytoskeleton as a result of increased hydrostatic pressure after the cell contracts [98]. 

#### 1.3.2. Composition

The composition of apoptotic bodies is in direct contrast with exosomes and MVs. Unlike exosomes and MVs, apoptotic bodies contain intact organelles, chromatin, and small amounts of glycosylated proteins [3,48,60,99]. Thus, one would expect to observe higher levels of proteins associated with the nucleus (i.e., histones), mitochondria (i.e., HSP60), Golgi apparatus, and endoplasmic reticulum (i.e., GRP78). Further, the proteomic profiles of apoptotic bodies and cell lysate are quite similar, whereas there are stark differences in the proteomic profiles between exosomes and cell lysate.

## 2. Isolation Methods

The potential benefits and uses of exosomes and other EVs in the clinical setting have been described above, however, a major hindrance in bringing exosomes into the clinical setting is the lack of standardization in isolation methods. Exosomes were originally isolated by ultracentrifugation-based methods, and while these methods remain the gold standard, other methods have been developed to address the challenges associated with ultracentrifugation [100,101]. These alternative methods have been developed based on isolation by size, immunoaffinity capture, and precipitation of exosomes, however, even these methods fail to exclusively isolate exosomes, and typically result in complex mixtures of EVs and other components of the extracellular space [1,2]. This is due to the complexity of biological fluids from which exosomes are being isolated from, the drastic overlap in the physiochemical and biochemical properties between exosomes and different EVs, and the heterogeneity among exosomes themselves [102,103]. Thus, the challenge remains to develop isolation techniques that can differentiate the different types of EVs in the extracellular matrix and do so rapidly, efficiently, reproducibly, and in a clinically friendly manner [5]. Further, the use of multiple isolation methods consecutively has been used to further enrich the exosomal content of a particular isolation, however, this also leads to increased cost, time, and technical training making it less clinically friendly [104]. An overview of some methods described in this review can be seen in Table 1. 

### 2.1. Ultracentrifugation Techniques

#### 2.1.1. Differential Ultracentrifugation

**Figure 1:**
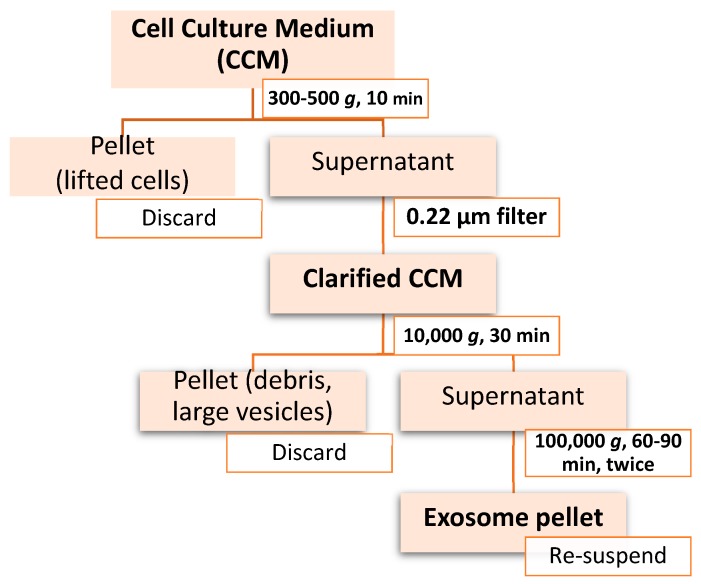
Workflow of differential ultracentrifugation for exosome isolation.

Differential ultracentrifugation was the first method used for exosome isolation and remains the gold standard for exosome isolation to date [100,110,111]. As is the case with all centrifugation methods, the separation of exosomes and other EVs from the extracellular matrix depends on density, size, and shape, with larger and more dense particles sedimenting out first [112]. A sample protocol for exosome isolation by differential ultracentrifugation is represented in the diagram in Figure 1. 

The 500× *g* step helps to pellet-out cellular debris and larger particles from the matrix. The 0.22 μm filtration and 10,000× *g* steps further purify the matrix, removing larger EVs and apoptotic bodies. Finally, the exosomes are pelleted out and washed in the 100,000× *g* centrifugation steps. The exosomal yield can be increased by using longer centrifugation times during the 100,000× *g* spins, however, it has been demonstrated that if >4 h is used, there is significant mechanical damage to the exosomes and higher levels of soluble protein contamination in the final preparation [106,113]. Even when less than 4 h is used during the 100,000× *g* spins, differential ultracentrifugation only results in an enrichment of exosomes, not a complete separation of exosomes from other components in the extracellular space [1]. In addition, differential ultracentrifugation is time consuming and requires large starting volumes (100s of mLs) of sample, making it difficult to process several biological samples in a short amount of time [1]. At the same time, however, differential ultracentrifugation requires little technical expertise, little to no sample pretreatment, and affordability over time since only one ultracentrifuge is needed for long term use [101].

#### 2.1.2. Density Gradient Centrifugation

Density gradient centrifugation is another ultracentrifugation method that is commonly employed in research settings. Like differential ultracentrifugation, separation is still based on size and density, however in density gradient centrifugation this occurs in the presence of a preconstructed density gradient, typically made of sucrose or iodoxinol, in the centrifuge tube [101,104]. The sample is placed at the top of the gradient, and when centrifugal force is applied, the particles in the sample pass through the gradient, which increases in density from top to bottom, at unique rates to allow for separation. The exosomes can then be collected by fractionation collection, typically in the density range of 1.1 and 1.2 g/mL [101,104]. Density gradient ultracentrifugation is very effective in separating EVs, including exosomes, from protein aggregates and non-membranous particles and is particularly useful for separating exosomes and other EVs from bodily fluids. However, like differential ultracentrifugation, it suffers from low exosome recovery [10,40,89,114]. Previous studies have demonstrated the coupling of differential ultracentrifugation with either Rate-Zonal Centrifugation or Isopycnic Centrifugation (2 types of density gradient ultracentrifugation) can drastically improve the purity and quality of the isolated exosomes, however, it requires additional time for gradient preparation and extra care during the acceleration and deceleration to prevent damage to the gradient [115].

##### Rate-Zonal Centrifugation

Rate-Zonal centrifugation allows for separation of particles based primarily on their sedimentation rate [115]. The sample containing EVs is placed on top of a shallow gradient and upon centrifugation the sample particles will separate into different zones based on their sedimentation rate as they move through a gradient with increasing density towards the bottom of the ultracentrifuge tube. The more dense particles will travel more quickly to the bottom of the tube as they can pass through the more dense layers easier than the smaller particles [115]. It is important to control the duration of centrifugation because eventually, since the particles are denser than the gradient, they will pellet at the bottom of the ultracentrifugation tube.

##### Isopycnic Centrifugation

In isopycnic centrifugation, particles sediment into the fraction of a steep density gradient with the same density, also known as the isopycnic position [115]. At this position, the gradient density is equal to the buoyant density of the particles, and the particles therefore remain in the given portion of the gradient [115]. In this method, the exosomes will remain at their unique isopycnic position and will not pellet out, no matter how long the centrifugation time lasts [115], which differs from Rate-Zonal centrifugation where particles will eventually pellet out due to the shallow density gradient used. Since apoptotic bodies, microvesicles, exosomes, and soluble proteins have different densities, their isopycnic positions will be at different levels of the gradient, providing a separation between the extracellular components.

### 2.2. Size Based Techniques

#### 2.2.1. Ultrafiltration

Ultrafiltration is one of the most common size-based techniques used for exosome isolation; the idea behind this method is the same as with conventional membrane filtration, where the separation of particles is based on the size and molecular weight cut off (MWCO) of the membrane being used [104]. That is, particles larger than the MWCO of the particular filter are retained by the filter and particles smaller than the MWCO of the filter are passed through the filter into the filtrate [101,104]. One challenge with ultrafiltration is the clogging and trapping of vesicles (and therefore loss of exosomes) on the filter unit [116]. While this can be minimized by starting with larger MWCO filters and moving to smaller ones, it leads to low isolation efficiency, and the exosomes lost on the membrane cannot be used in downstream analysis [101]. While ultrafiltration is less time consuming than ultracentrifugation and requires no special instrumentation, it can still lead to particle deformation and lysis of exosomes due to the shear force, though this can be reduced by monitoring and regulating transmembrane pressure [106]. 

#### 2.2.2. Exosome Isolation Kit

A commercially available isolation kit, the ExoMir Kit (Bioo Scientific; Austin, TX, USA) has been developed to isolate exosomes based on size. Essentially, two membranes (200 nm and 20 nm) are placed into a syringe with the 200 nm filter at the top and the 20 nm filter at the bottom. The sample is typically pretreated with a low speed centrifugation, to pellet cells and cellular debris, and proteinase K, to help breakdown larger particles and prevent the membrane from clogging. After pretreatment, the sample is passed through the syringe where the larger vesicles (>200 nm) remain above the first filter, the smaller vesicles (<200 nm and >20 nm) remain between the two filters in the syringe, and the smallest vesicles (<20 nm) are passed through the syringe and discarded. Other methods relying on the same general principle have also been developed, such as the ExoTIC technology, in order to make the isolation of exosomes a more clinically friendly procedure [117].

#### 2.2.3. Sequential Filtration

The idea behind sequential filtration for exosome isolation is similar to the ExoMir Kit or ExoTIC methods discussed in Section 2.2.2, in that it relies on a series of filtration steps for exosome enrichment. In sequential filtration, the initial steps involves filtration with a 100 nm filter to eliminate cells, cellular debris, and large rigid particles [118]. Particles that are larger than 100 nm in diameter, such as exosomes and microvesicles, are able to pass through the 100 nm filter as long as they are soft and flexible [118]. However, the more rigid components associated with cellular debris are filtered away [118]. The filtrate then undergoes tangential flow filtration with a 500 kDa MWCO membrane to remove soluble proteins and other contaminants [118]. Finally, concentrated retentate is then filtered with a 100 nm track-etch filter for exosome enrichment [118]. The primary advantages of this methodology are that it can isolate exosomes from 150 mL of media within a day, is automatable, and produces intact and biologically active exosome material, some of which have been used in clinical trials [118,119,120].

#### 2.2.4. Size Exclusion Chromatography (SEC)

The use of size exclusion chromatography (SEC) to isolate exosomes from other EVs based on size is performed the same way as if one wanted to separate proteins of different sizes. That is, a column is packed with a porous stationary phase in which small particles can penetrate. This penetration slows down the movement of the smaller particles through the tube, causing them to elute later in the gradient, after the larger particles. Typically, size exclusion is used in parallel to ultracentrifugation methods, where the exosome pellet is re-suspended after enrichment by ultracentrifugation and then further purified using SEC [121,122]. While SEC methods preserve vesicle structure, integrity, and biological activity, they require run times of several hours, are not easily scalable, and cannot be used for high throughput applications [122]. However, iZON science has produced a qEV Exosome Isolation Kit, which allows for rapid, cost effective, high precision exosome isolation within 15 min based on the SEC methodology [123]. Their products allow for exosome isolation from <150 μL up to 10 mL volume of starting material with porous resins of 35 nm or 75 nm for optimal exosome isolation [123]. Development of such methodologies may bring about standardization in the area of exosome isolation, making the use of exosomes in the clinical setting more realistic.

#### 2.2.5. Flow Field-Flow Fractionation (FFFF) 

Flow Field-Flow Fractionation (FFFF) is a new technique used to isolate exosomes based on size. In this method, the sample is injected into a chamber and subjected to parabolic flow as it is pushed down the length of the chamber [124]. At the same time, a crossflow (a flow perpendicular to the parabolic flow) is used to create the separation of the particles in the sample [124]. Larger particles are more affected by the crossflow, so they are pushed closer to the walls of the chamber, where the parabolic flow is slower [124]. Thus, the larger particles elute after the smaller particles, which are less affected by the crossflow, remain in the center of the parabolic flow, and elute earlier [124].

#### 2.2.6. Hydrostatic Filtration Dialysis (HFD)

In traditional dialysis, separation of particles in the sample is achieved by diffusion of particles across a porous membrane. The selectivity of the separation is dependent on the MWCO of the given membrane; particles smaller than the MWCO of the membrane will diffuse across the membrane, and particles larger than the MWCO of the membrane will remain on the starting side of the membrane. In HFD, the sample is forced through a dialysis tube with a MWCO of 1000 kDa by hydrostatic pressure. The solvent and small solutes pass easily through the tube and the larger particles, such as exosomes and other EVs, remain in the tube, where they can be collected [125]. Typically, ultracentrifugation methods are used after HFD isolation to further separate exosomes from other EVs retained in the dialysis tube [125]. 

### 2.3. Immunoaffinity Capture-Based Techniques

Immunoaffinity capture-based techniques rely on the use of an antibody to capture exosomes based on the expression of the antigen on the surface of the exosome. Antibodies for a specific antigen of interest can be attached to plate (ex. ELISA, see Section 2.3.1 below), magnetic beads (see Section 2.3.2 below), resins, and microfluidic devices (see Section 2.5 below) [101]. A major benefit of these techniques over others is that it allows for isolation of exosomes derived from a specific source [10,126,127]. For example, a well-established hepatocellular protein marker is Asialoglycoprotein receptor 1 (ASGR1). The presence of this protein has been established in hepatocyte derived exosomes [128], and therefore has the ability to be used as a marker to isolate liver derived exosomes. Not only do immunoaffinity methods have the potential to aid in the isolation of a specific sub-set of exosomes from a complex mixture, they also have the potential to separate exosomes from other types of EVs, should a specific marker for exosomes be identified and agreed upon [106]. The main limitation in developing this method is that the protein/antigen used to capture the exosomes must be expressed on the surface of exosomes, since the antibody will not be able to capture an antigen enclosed within the vesicle [104]. In addition, the specificity of the assay is limited to the specificity of the antibody used, however, it has been well demonstrated that immunoaffinity methods result in lower yield of isolated exosomes with higher purity than methods that isolate exosomes based on other properties [129]. Due to the complexity of biological fluids, such as plasma, immunoaffinity capture-based techniques are often used after exosomal enrichment by ultracentrifugation or ultrafiltration [126].

#### 2.3.1. Enzyme-Linked Immunosorbent Assay (ELISA)

An ELISA based assay, in which an antibody against an antigen of interest is immobilized on the surface of a microplate, is one type of immunoaffinity capture-based technique that is used to isolate exosomes from a sample. The exosome sample is exposed to the well containing the immobilized antibody, and the exosomes expressing the antigen are now immobilized onto the plate due to the antibody-antigen interaction. The un-captured exosomes and sample contents are washed away, and the immobilized exosomes can be detected using another antibody containing an absorbent tag. In the EV field, ELISA has been used to isolate exosomes from urine, plasma, and serum, and can even be quantitative when standards (of known exosome amounts) are used to create a calibration curve. This method has been around for many years and is currently used in the clinical setting to test a patient’s blood for different antibodies against different infectious diseases, such as HIV, Zika, Lyme Disease, and others [130]. However, it is yet to be used in the clinical setting for exosome applications due to the required sample pretreatment by ultracentrifugation or ultrafiltration.

#### 2.3.2. Magneto-Immunoprecipitation

In the case of magneto-immunocapture, a biotinylated antibody against the antigen of interest is attached to the surface of streptavidin coated magnetic beads. The antibody coated beads are then incubated with the sample from which exosomes are to be isolated from. The major benefit of this method over ELISA is that the beads provide a larger surface area for capturing exosomes, leading to higher isolation efficiency. Additionally, there is no upper limit of sample starting volume when using the magnetic beads, whereas the microplate-based ELISA assay has a maximum sample volume of 100 μL that can be held within the well of a typical 96-well microplate. Not only does magneto-immunocapture provide better isolation efficiency and is capable of handling large sample volumes, the exosomes captured on the beads can be eluted and used for downstream analysis. When comparing magneto-immunocapture to the gold standard exosome isolation method, ultracentrifugation, magneto-immunocapture leads to a more pure exosome preparation, is quicker, and requires no advanced or expensive instrumentation [104]. Additionally, the magneto-immunocapture methodology is better for preserving the activity of exosomal proteins than other isolation methods, such as ultracentrifugation or ultrafiltration [131].

### 2.4. Exosome Precipitation

#### 2.4.1. Polyethylene Glycol (PEG) Precipitation

Precipitation of exosomal vesicles is typically done by introducing a water excluding polymer, such as polyethylene glycol (PEG), into the sample. The PEG polymer then “ties-up” the water molecules, causing other particles, such as exosomes to precipitate out of solution [106]. The precipitated vesicles can then be pelleted by centrifugation and used for different downstream analysis [106]. This isolation method is quick, simple, requiring little technical expertise or expensive equipment [101,104]. Additionally, it can be used for a variety of starting volumes (from 100 μL up to several mLs) and therefore is suitable for use in various research and clinical settings [101,104]. However, the major drawback with this methodology, and the reason it cannot be immediately employed in the clinical setting is due to the lack of selectivity [106,129]. Not only do the PEG polymers cause precipitation of exosomal vesicles, they also causes precipitation of other extracellular vesicles, extracellular proteins, and protein aggregates [100]. Therefore, it is important to include some sample pretreatment, such as filtration and/or ultracentrifugation, before using such methods in order to reduce contamination of final exosomal preparation [104]. Several commercially available kits have been produced based on exosome precipitation for isolation of exosomes from cell culture medium and a variety of bodily fluids, e.g., ExoQuick by System Biosciences (Palo Alto, CA, USA) and Total Exosome Isolation Kit by Thermo Fisher Scientific [132].

#### 2.4.2. Lectin Induced Agglutination

An alternative to PEG precipitation is lectin precipitation. Lectins are a family of proteins that bind carbohydrate moieties of other particles at a very high specificity. This is able to aid in exosome isolation when lectins bind to carbohydrates on the surface of exosomes. When lectins bind to the carbohydrates on the surface of exosomes, it alters their solubility, causing them to precipitate out of solution. Typically, the sample is pretreated by ultracentrifugation to remove any cellular debris or other components that may also contain carbohydrates. The sample is then incubated overnight with the lectin, for example Concanavalin A or Phytohemagglutinin at 1 mg/L, and the precipitated exosomes can then be pelleted using centrifugation [133]. Like PEG precipitation methods, the lectin precipitation methods are straightforward requiring little time and expertise, however the co-precipitation of other soluble components is negligible unless they are highly glycosylated.

### 2.5. Microfluidic Based Isolation Techniques

Microfluidic based exosome isolation methods have been developed in order to address issues with more traditional methods, and make the use of exosomes in the clinical setting more feasible. The primary advantage of microfluidic techniques is that they have the ability to isolate exosomes based on their physical and biochemical properties simultaneously [104]. Additionally, microfluidic isolation methods typically are rapid, efficient, require small starting volumes (10 s–100 s of μL), and allow for the development of innovative separation mechanisms such as acoustic, electrophoretic, and electromagnetic properties of the exosomal vesicles [109,134].

#### 2.5.1. Acoustic Nanofilter

Acoustic nanofilter is a microfluidic isolation technique in which exosomes and other EVs are separated from the matrix based on size. The matrix, containing exosomes, EVs, and other extracellular components is injected into a chamber where it is exposed to ultrasound waves. These waves exert radiation forces onto the particles, and the particle’s response to these forces is dependent upon its size and density [109]. Specifically, larger particles experience stronger radiation forces and therefore migrate faster towards the pressure nodes [109]. The ultrasonic waves can be tuned in such a way to separate particles above and below any desired size [109]. While this specific methodology is still in development stages, its simplicity, quickness, tunability, and low starting volume (50 μL) of material make it a promising tool for potential use in the clinical setting.

#### 2.5.2. Immuno-Based Microfluidic Isolation

The principles behind immuno-based microfluidic isolation techniques are very similar to those of ELISA (Section 2.3.1). The isolation of exosomes is based on an interaction between a membrane bound protein on the exosomal vesicle and an antibody against the protein which is immobilized on a microfluidic chip. The primary advantage of this technique over ELISA is that exosomes have been isolated from as little as 10 s–100 s of μL of serum in 60 min, whereas ELISA assays require prior isolation of exosomes (via ultracentrifugation, ultrafiltration etc.) from the plasma or serum [108,135]. Much like ELISA, the specificity of the assay is dependent on the specificity of the antibody used. A commercially available product, ExoChip, has been developed for isolation of exosomes using the microfluidic technology. This product has an anti-CD63 antibody immobilized on the surface of the chip. The CD63 protein is considered an exosomal marker protein and has been found to be expressed in exosomes from many cell types, and thus allows for isolation of exosomes from a sample matrix regardless of the cell source [107]. Other microfluidic based isolation methodologies have been developed, for example the ExoSearch Chip, which has demonstrated the ability to isolate exosomes from as little at 20 μL of plasma in 40 min [108]. Unlike the ExoChip, the ExoSearch Chip allows for isolation of specific subpopulations of exosomes of interest, assuming that the antigen used to differentiate the subpopulation of exosomes is expressed on the surface of the exosomes, and can be recognized by the immobilized antibody on the beads [108]. The development of the microfluidic based technologies is essential to bringing the diagnostic, therapeutic, and prognostic capabilities of exosomes to the clinical setting because in comparison to all other isolation methods, these methods require the smallest amounts of plasma/serum, least amount of time, and are most cost efficient and require minimal expertise and training.

## 3. Analysis of Exosomes

Initially, isolated extracellular vesicles were characterized primarily by their protein concentration [1]. However, the protein concentration of isolated EVs is typically overestimated due to contamination, and does not take into consideration the different protein profiles that can vary between different subtypes of EVs [1]. Thus, as the uses of EVs became more prevalent and of interest, they were studied by more sophisticated methods. Today, there are typically two different types of analysis performed on the isolated vesicles, that is physical and chemical/biochemical/compositional analysis. Physical analysis, which gives insight to particle size and/or concentration, is done using nanoparticle tracking analysis (NTA), dynamic light scattering (DLS), electron microscopy, and tunable resistive pulse sensing (tRPS). The chemical/biochemical/compositional analysis is typically done via staining, immunoblotting, or proteomic analysis, and gives information regarding the content of the isolated vesicles. A major challenge in this area is developing methodologies that can differentiate the different types of EVs, are easily standardized, and well multiplexed. What makes this the most difficult is the fact that the proteomic profiles of exosomes are changed when different isolation methods are used to isolate exosomes from the same cell line [2]. This section is an overview of the current methods used for exosomal protein analysis and, unless otherwise noted, required some sort of isolation or enrichment of exosomal vesicles (see Section 2 of this paper) prior to analysis.

### 3.1. Physical Analysis

#### 3.1.1. Nanoparticle Tracking Analysis (NTA)

Nanoparticle tracking analysis, or NTA, allows for the determination of both particle size and concentration. The size of the particles is estimated using the Stokes–Einstein equation, where the diffusion coefficient is based on the Brownian motion of particles within the chamber. The laser light is scattered as it interacts with the particles (under Brownian motion) within the chamber, and the scattered light is collected by a microscope that has a camera mounted to it [136]. The camera on top of the microscope captures the movement of particles in a video, and then the NTA software uses the movement of the particles in the video to estimate the particle size and concentration [136]. NTA is capable of determining particle size between 10 and 1000 nm in diameter, which is within the size of exosomes which are known to be between 50–150 nm [5,137]. The challenge with NTA, however, is that it requires sample volumes of ~0.5 mL, and optimization of data collection and analysis parameters [138,139].

#### 3.1.2. Dynamic Light Scattering (DLS)

Like NTA, dynamic light scattering, or DLS, uses the scattered light due to Brownian motion of particles to estimate particle size and concentration. However, instead of using the scattered light to determine the particle’s diffusion coefficient, DLS uses the fluctuations in the intensity of the scattered light to estimate the particle’s size [138,139,140]. Unlike NTA, DLS requires very little sample volume (70 μL) and is easy to use, with few parameters needed for optimization [138,139]. While DLS has its benefits over NTA, its major drawback is in the analysis of heterogenous mixtures. Specifically, the intensity of scattered light is proportional to the sixth power of particle diameter, making the scattered light due to smaller particles harder to detect, thus it often produces data that is skewed towards larger particle sizes when there is a mixture of particle sizes present in the suspension [138,139]. Therefore, NTA is best for differentiation of heterogenous populations of particles [139].

#### 3.1.3. Electron Microscopy

The two common types of electron microscopy used to assess the morphology of exosomal vesicles are transmission electron microscopy (TEM) and scanning electron microscopy (SEM). Both SEM and TEM produce high resolution images of submicron particles using a beam of electrons. The difference between the two is which electrons are detected. Simply put, in SEM, the scattered electrons are detected, and in TEM, the electrons that pass through the sample are detected [141]. More specifically, in SEM, the electrons are scattered when they interact with the particles in the sample. The scattered electrons are then captured and detected, which produces this image of particles. In TEM, however, the electrons that do not interact with the particles pass through the sample and are detected using a fluorescent screen. The particles of the sample create dark areas, or shadows, on the fluorescent screen thus producing an image [141]. In the case of exosomes, both TEM and SEM demonstrate similar size distribution of particles but slightly different morphologies [142]. That is, in TEM and SEM, the exosomal vesicles typically have a divot in their center. This is likely due to the drying process associated with the sample preparation required for TEM and SEM [142].

#### 3.1.4. Tunable Resistive Pulse Sensing (tRPS)

Tunable resistive pulse sensing, or tRPS, is another technique that can be used in order to get the size distribution and concentration of particles in a sample. Essentially, a fluid cell is divided in half by a non-conductive nano-membrane [143]. One half of the cell contains the suspension and the other half contains a particle free electrolyte [143]. A potential is applied across the two cells and the particles then flow from their half, through the nano-membrane, and to the other half. As the particles cross the membrane, however, it causes a disruption, or resistive pulse, in the current across the two different cells [143]. The length of the resistive pulse can be correlated to the size of the particle producing that particular resistive pulse, if a series of standards with known diameters are used to build the calibration curve [143]. In addition, the number of resistive pulses can be measured over a given time (the rate of resistive pulses), which reveals information regarding particle concentration within a sample [143].

### 3.2. Chemical, Biochemical, and Compositional Analysis

#### 3.2.1. Immunodetection Methods

Immunodetection methods are analysis methods that rely on the recognition of a polyclonal, or monoclonal, antibody to its antigen in the sample. Such methods are commonly employed in biomedical research laboratories and are often used to establish the purity of isolated EVs by observing the presence or absence of marker proteins, as well as detecting target proteins of interest.

##### Flow Cytometry

Flow cytometry is often considered a physical form of analysis since it allows for visual observation of exosomes, however, it requires some knowledge regarding the protein composition of the exosomal vesicles in order for the vesicles to be detected, thus is also considered a form of compositional analysis. While the flow cytometry technology is quickly advancing, with newer instruments having detection limits as low as 100–200 nm, most instruments have a 300–500 nm limit of detection, which is much larger than the size of exosomal vesicles [144,145]. The challenge of flow cytometry in the field of EVs is that, despite the recent advances, it requires a single particle suspension which can be very challenging to achieve when the exosomal concentration is high, or if aggregation of exosomal vesicles occurs during the isolation process [145]. Aggregation of vesicles results in the observation of multiple particles at a single time which results in inaccurate data [145]. Thus, it requires the immobilization of exosomes on the surface of beads (either by immunocapture or covalent conjugation) in order to be observed by the flow cytometer. Once exosomes are immobilized on the surface of the beads, the exosomal vesicles are exposed to a fluorescently conjugated antibody against an antigen that is known/expected to be expressed on the exosomal surface [145]. The exosomal vesicles conjugated to the beads and the fluorescent antibody can be viewed under an epifluorescent microscope (EPI) prior to flow cytometry. Then, as the sample passes through the laser of the flow cytometer, it emits a fluorescent signal which is detected [144,145]. Not only does this allow for high throughput analysis of exosomes, it also allows for quantification or classification of exosomes based on the antigen expression [145].

##### Western Blotting

The principles behind immunoblotting, or Western blotting, involve the affinity binding of an antigen (target proteins) and an antibody that specifically recognized the antigen. Unlike flow cytometry, Western blotting does not allow for observation of intact vesicles, rather, the vesicles are lysed and the proteins are denatured and reduced during the sample preparation [146]. After denaturation, the proteins are separated by SDS-PAGE and then transferred to a nitrocellulose or polyvinylidene fluoride (PVDF) membrane. The remaining open pores on the membrane are filled with protein (from non-fat milk) and/or detergent and then exposed to an antibody against an antigen of interest. The antibody ideally specifically recognizes the antigen on the surface of the membrane. The membrane is then exposed to a secondary antibody, which is an antibody against the species of the initial (primary) antibody used to recognize the antigen. The secondary antibody is detected due to its fluorescent tag, or by the horseradish peroxidase/alkaline phosphatase group coupled to the secondary antibody. The Western blotting methodology is among the most commonly used analysis methods for analysis of exosomes due to its ease of use, wide accessibility, and the ability to detect exosomal surface proteins and internal proteins. The primary pitfall, however, is that it is not well multiplexed and the specificity and reproducibility are limited by the quality of the antibody used. The lack of multiplicity results in the use of a large amount of exosomal protein used to gain a minimal amount of information. Since the collection and isolation of exosomes is often a time-consuming process with low yield, more multiplexed analysis methods would be highly beneficial.

##### Integrated Immuno-Isolation and Protein Analysis of Exosomes 

A novel microfluidic assay has been developed that allows for not only isolation, but also for protein analysis of exosomal vesicles. As discussed above in Section 2.5 (immuno-based microfluidic isolation) microfluidic devices are a developing technology, which may be key to bringing exosomes to use in the clinical setting. Many of the existing microfluidic techniques allow for detection of exosomes using fluorescent antibodies against an antigen of interest on the surface of exosomes, this time taking place on the surface of a chip rather than a membrane or magnetic bead. However, the novel device described in [135] allows for isolation of the exosomal vesicles, on a microfluidic chip, but then introduces a lysis buffer to lyse the captured exosomal vesicles. The lysate is then eluted from the microfluidic chip, and the biomarkers of interest can be probed for, independent of whether or not the biomarker is contained within the vesicles or on the surface of the exosomal vesicle. This allows for a broader spectrum of antigens to be detected and allows for the development of biomarkers within exosomes to be used rather than those only on the exosome surface.

#### 3.2.2. Thermophoretic Profiling

This methodology is similar to the integrated immune-isolation discussed above (3.2.1 Integrated Immuno-Isolation and Protein Analysis of Exosomes), in that it isolates, or enriches, the vesicles while giving some compositional information at the same time. The difference in thermophoretic profiling compared to the integrated immune-isolation, is that is does not rely on the use of antibodies. Instead, <1 μL of serum is diluted 10× into phosphate buffered saline (PBS) and incubated with seven different fluorescently conjugated nucleotide aptamers, which specifically target different proteins on the surface of exosomes in the serum [147]. The aptamer-exosome incubation takes place for 2 h at room temperature, at which point the chamber is exposed to a 1480 nm laser for 10 min. This process drives the exosomal vesicles to the center of the laser point, leading to accumulation of the vesicles, which can then be investigated for presence/absence of specific proteins based on the fluorescent detection of the EV conjugated aptamers. The authors demonstrated lack of fluorescent signal without the laser heating, and also that free aptamers and small serum proteins could not be enriched when exposed to the laser [147]. Such methodologies, which use very little serum, do not require any sample pretreatment or time-consuming exosome isolation, are reliable, reproducible, specific, do not require high technical expertise or training, and give information regarding the presence of cancer biomarkers in the EVs within hours, are methods that could make the use of exosomes in the clinical setting a reality.

#### 3.2.3. Mass Spectrometry (MS)-Based Proteomic Analysis

##### Global Proteomic Approaches

Global proteomics is a method used to identify as many proteins as possible within a sample. This can be done two different ways, via data dependent acquisition (DDA) or data independent acquisition (DIA), with DDA being used more commonly than DIA [148]. In DDA experiments, a survey MS spectrum is collected, and the most abundant ions are then selected for fragmentation and MS/MS analysis. Thus, the data depend on the abundance of the ion in the survey MS spectrum relative to other ions eluting at the same retention time in the same MS spectrum. The tandem MS data are processed using software (for example Mascot) to get information on the amino acid sequence which can then be used to identify the proteins present in a sample. In DIA experiments, ions are not selected based on abundance for fragmentation, but rather it is an attempt to fragment and get MS/MS data on all ions within a given mass range. In these experiments, fragmentation libraries are used to sort the mixed MS/MS data and identify the proteins present within the sample. Both DDA and DIA experiments can be done with top-down sample preparation and bottom-up sample preparation. The top-down approach, which is when no proteolysis of the proteins takes place prior to MS analysis, remains challenging from a technological perspective [149]. While advances have been made in the ability to separate and fragment intact proteins, the bottom-up approach remains the most commonly used sample preparation method. In the bottom-up approach, the sample is digested with a protease, such as trypsin or pepsin, prior to MS analysis. The smaller protein fragments (peptides) produced by the enzymes are easier to separate, ionize, and fragment for high quality MS/MS data. However, the versatility of this technique is limited because the MS/MS data collection occurs after fragmentation by collision induced dissociation (CID). During CID, the weakest bonds are broken first, and these bonds are typically bonds associated with PTMs, thus making the PTM analysis of these peptides difficult. The use of top-down approaches, however, would reveal information regarding a protein’s PTMs as the MS/MS data are collected after electron-transfer dissociation (ETD), which causes fragmentation of the peptide/protein backbone while leaving PTMs intact. Additionally, top-down approaches would reveal sequence variations in proteins between the exosomes and parent cells, which may be useful in further understanding the role of specific proteins within exosomal vesicles. Typically, global proteomic experiments on exosomes result in several hundred to several thousand proteins identified, dependent upon the amount of starting material used, the sample preparation method, and the algorithm used for data analysis. The use of global proteomics in the field of exosomes is often for identification of novel biomarkers for different cancers or diseases, but sometimes the presence of the protein (or biomarker) is also present in exosomes from healthy tissues. Thus, it is not only important to identify the proteins present in the exosomal vesicles, but also be able to quantify the proteins present within the exosomal vesicles. Protein quantification in global proteomics can be done with labeled techniques, such as SILAC or iTRAQ, which involve the incorporation of a stable heavy isotope labeled amino acid into the peptide of interest [150]. However, not only is this an expensive process, but the peptide of interest may not always be known. Thus, label free techniques have been developed in order to quantify the proteins identified in DDA or DIA experiments. The techniques rely either on the peak area of the parent ion or the spectral count, which is the number of times a specific peptide is selected for fragmentation in a data dependent LC-MS/MS [151]. While both of these methods require some sort of normalization, they are now used more frequently than the labeling techniques. The use of global proteomics in the field of EVs, and specifically exosomes, has aided, and continues to aid, in the development of biomarkers for different diseases and cancers. Further, there is potential for this technique to reveal the purpose and activity of different proteins in the exosomal vesicles, and how they are similar and different to those in the parent cells, as the technology around the top-down methodology continues to develop.

##### Targeted Proteomic Approaches

As opposed to global proteomics, where the goal is to identify as many proteins as possible in a sample, targeted proteomic analysis is used to identify and quantify a predefined set of proteins in a given sample [148]. The most common targeted proteomic approach is multiple reaction monitoring, or MRM. Due to upper mass limitations of triple quadrupole instruments, used for MRM methods, the bottom-up approach must be used and specific peptides for each protein of interest must be selected prior to analysis. That is, a peptide generated by the trypsin or pepsin digestion must be selected and be unique for the protein that is to be monitored [152,153]. Once the peptide is selected, transitions, or fragments, of the peptide can be established and detected by the instrument. Essentially, in the first quadrupole (Q1) of a triple quadrupole instrument, the parent ion, which is the intact peptide selected to represent the protein of interest, is selected to pass through into the second quadrupole (q2). All other ions are filtered out in Q1 quadrupole and do not pass into q2 [148]. Once in q2, the parent ion will be fragmented and then passed into the third quadrupole, Q3. In Q3, a specific fragment ion is selected to reach the detector and all other fragment ions are filtered out and do not reach the detector. By monitoring multiple unique peptides for a single protein, and then multiple fragments for each peptide, the specificity and accuracy of the method can be greatly enhanced. Typically, it is recommended that at least 2 unique peptides are used to monitor a specific protein, and at least three transitions are used to monitor each peptide, thus a total of 6 signals are used to monitor a single protein of interest. Additionally, the MRM method is extremely well multiplexed, as long as the chromatography allows for good separation of the peptides. By monitoring different peptides at different retention times, the MRM method can give highly sensitive, specific, and quantitative information on hundreds of peptides in a single experiment. Absolute quantification for proteins (via peptides) in a sample can be assessed using the MRM methodology by spiking stable isotope labeled peptides into the sample prior to analysis. Relative quantification can be done using simple normalization techniques, similar to the normalization done in the label free global proteomic approaches. However, because MRM methods have lower limits of detection, greater dynamic ranges, and increased specificity, it is the mass spectrometry proteomic approach of choice for the rapid identification and quantification of a predetermined set of proteins in a sample [148]. In the realm of EVs, specifically exosomes, the development of MRM methods to characterize isolated exosomes would be beneficial not only due to its multiplicity and specificity over the traditional Western blot methodology, but also because a predefined set of proteins (exosomal marker proteins and non exosomal marker proteins) have been described.

## 4. Conclusions

**Figure 2:**
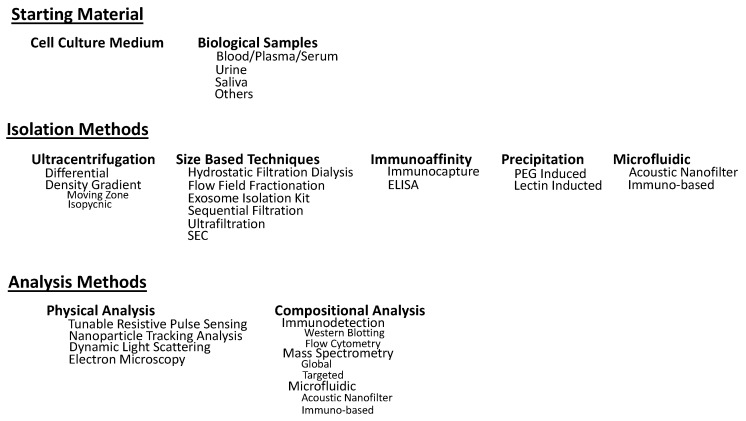
Overview of isolation and analysis covered in this review.

The uses of EVs in the clinical setting for diagnostic, prognostic, therapeutic, and drug delivery tools has well been demonstrated and continues to be a subject of intense study simply based on the ever-growing literature on the topic. Each isolation and analysis method (see Figure 2 for review) has its own set of benefits and drawbacks, and it has been demonstrated that different isolation methods used to isolate exosomes from the same cell type results in different proteomic profiles, further complicating the situation. Therefore, instead of focusing on establishing a set of exosomal/non exosomal marker proteins secreted by all cell types independent of the isolation method, it may be more beneficial to focus on the development of exosomal/non exosomal marker proteins for a given cell type, independent of isolation method, or a set of exosomal/non exosomal marker proteins for all exosomes, regardless of their origin, when isolated by a specific method. Regardless, it is of utmost importance to consider and incorporate the recommendations from the Minimal Information for Studies of Extracellular Vesicles 2018 (MISEV2018) [154] when conducting and reporting EV-related works to improve rigor and reproducibility of identified EV markers. 

## Figures and Tables

**Table 1:** Comparison of exosomal isolation techniques based on recovery, purity, required sample volume, and time required for isolation.

Isolation Technique	Recovery	Purity	Sample Volume	Time Required	Reference
Ultracentrifugation	5–25%	Low	100s of mLs	8 h	[10]
Density Gradient	Higher than UC	Similar to UC	up to 1 mL	20 h	[105]
Precipitation Kits	N/A	Low	>100 μL	Overnight	[106]
ExoChip	N/A	N/A	<400 μL	<2 h	[107]
Immunoprecipitation	>99% bead recovery	Higher than UC	up to 1 mL	Overnight	[10]
ExoSearch Chip	42–97%	Higher than UC	20 μL	40 min	[108]
Acoustic Nanofilter	>80%	High	50 μL	<30 min	[109]

N/A: not available.
